# TELE-REHABILITATION USING TRANSCRANIAL DIRECT CURRENT STIMULATION COMBINED WITH EXERCISE IN PEOPLE WITH SPINAL CORD INJURY: A RANDOMIZED CONTROLLED TRIAL

**DOI:** 10.2340/jrm.v57.42353

**Published:** 2025-05-07

**Authors:** Thanwarat CHANTANACHAI, Irin APIWORAJIRAWIT, Pipat KLAMRUEN, Benchaporn ANEKSAN, Paradee AUVICHAYAPAT, Alexandra LACKMY-VALLÉE, Wanalee KLOMJAI

**Affiliations:** 1 Faculty of Physical Therapy, Neuro Electrical Stimulation laboratory (NeuE), Mahidol University, Nakhon Pathom, Thailand; 2 Sirindhorn National Medical Rehabilitation Institute, Nonthaburi, Thailand; 3 Department of Physiology, Faculty of Medicine, Khon Kaen University, Khon Kaen, Thailand; 4 Sorbonne Université, CNRS, INSERM, Laboratoire d'Imagerie Biomédicale, LIB, Paris, France

**Keywords:** spinal cord injuries, telerehabilitation, transcranial direct current stimulation, neurological rehabilitation, exercise

## Abstract

**Objective:**

This study explored the effects of home-based transcranial direct current stimulation combined with exercise on motor and sensory function, spasticity, functional and transfer performance, and quality of life.

**Design:**

A prospective, double-blind, randomized, sham-controlled trial.

**Subjects and methods:**

Thirty individuals with SCI were allocated to receive either active transcranial direct current stimulation or sham transcranial direct current stimulation, followed by the same tele-rehabilitation programme, for 12 sessions over 4 weeks (3 sessions/week). Each session included 20 min of transcranial direct current stimulation followed by 1 h of tele-supervised exercise. Primary outcome was the International Standards for Neurological Classification of Spinal Cord Injury (ISNCSCI). Secondary outcomes included (*i*) the upper limb muscle strength evaluated by hand-held dynamometer, (*ii*) spasticity evaluated by H reflex and modified-Modified Ashworth Scale, (*iii*) functional performance assessed by the spinal cord independence measure III, (*iv*) transfer performance assessed by the transfer assessment instrument, and (*v*) quality of life assessed by WHOQOL-BREF. Outcomes were assessed at baseline, post-intervention, and 1-month follow-up.

**Results:**

Two-way mixed ANOVA revealed an interaction effects between group and time (F_(1,18)_=4.49, p=0.043) and main effects of time (F_(1,18)_=7.82, *p=*0.009). Bonferroni post-hoc analysis showed a significant improvement only within the active group at 1-month follow-up (*p=*0.002) for the upper extremity motor scores (UEMS). No significant differences were observed for any of the secondary outcomes.

**Conclusion:**

The effect of 12 sessions of home-based transcranial direct current stimulation combined with exercise was limited to improved upper limb motor recovery, with after-effect at 1-month post-intervention as compared with exercise alone. No improvements were found in sensory function, spasticity, functional and transfer performance, and quality of life. However, this intervention appeared to be feasible, safe, and well-adhered to and provides insight into the use of transcranial direct current stimulation as a tool for tele-rehabilitation in a spinal cord injury outpatient population.

## LAY ABSTRACT

This study explored whether home-based telerehabilitation using transcranial direct current stimulation combined with exercise could help to improve motor and sensory recovery, spasticity, functional mobility, and quality of life in people with spinal cord injury. We tested this approach in 30 participants who were divided into 2 groups: 1 received real transcranial direct current stimulation (15 participants), while the other received sham (placebo) stimulation (15 participants). Both groups received transcranial direct current stimulation (real or sham) followed by 1 h of supervised exercises by a physical therapist via video call, 3 times a week for 4 weeks (in total 12 times). The results showed that participants who received real transcranial direct current stimulation showed better upper limb motor function, and this improvement lasted for a month after treatment. However, there were no changes in other outcomes. Overall, transcranial direct current stimulation combined with exercise at home was safe and feasible for people with spinal cord injury, but its benefit was mainly limited to improving the upper limb motor function.

Rehabilitation for individuals with spinal cord injury (SCI) is an essential prerequisite for reclaiming functional independence and enhancing the overall quality of life (1). However, acute hospital and inpatient rehabilitation stays are becoming shorter, limiting patients and families in achieving maximum independence post-SCI (2). Upon discharge home, many face barriers such as economic constraints, transportation issues, and remote locations hindering access to specialty clinics (3). Tele-rehabilitation has emerged as a strategy to overcome these barriers, enabling remote delivery of rehabilitation services (4). Tele-rehabilitation has been shown to improve functional activity and daily living activity in individuals with SCI (5, 6). However, achieving full functional independence requires prolonged rehabilitation, and conventional inpatient rehabilitation alone may not be sufficient to support recovery (7). Therefore, tele-rehabilitation combined with advanced therapeutic techniques may help address these challenges and support individuals with SCI in achieving optimal recovery and independence.

Neuromodulation therapy using transcranial direct current stimulation (tDCS) is adjunctive treatment in neurorehabilitation to promote motor and sensory recovery after SCI. The proposed mechanisms underlying its effect may include enhancing residual descending connections (8–10) and inducing spinal plasticity (9). Previous studies have indicated that, within certain dose limits, anodal tDCS increased cortical excitability, while cathodal stimulation decreased it (11, 12). Moreover, anodal tDCS applied over the primary motor cortex has been demonstrated to modulate spinal network excitability (13). Additionally, using tDCS at home is a safe, accessible, convenient, and scalable treatment option for people with neurological deficits (14, 15). Earlier studies primarily focus on the effects of tDCS on pain outcomes in SCI. Meta-analytic results indicate a moderate effect of tDCS in reducing neuropathic pain among individuals with SCI (16). Research on its effects on motor outcomes has gained increasing attention more recently. A meta-analysis revealed that anodal tDCS, when combined with motor training such as massed practice, gait training, and robotic training, significantly improved motor function compared with sham tDCS in individuals with incomplete SCI (17). However, to date, no evidence exists on combining tDCS with tele-rehabilitation exercise programmes in an SCI population, despite being suggested as an add-on in intervention in neurorehabilitation, and with a possible option for home use.

This study aimed to explore the effect of 12 sessions (3 times/week for 4 weeks) of home-based active tDCS combined with an exercise programme compared with home-based exercise alone (sham group) on motor and sensory function, spasticity, functional and transfer performance, and quality of life in individuals with SCI. The hypothesis of this study was that active tDCS combined with exercise would lead to greater improvements in these outcomes compared with the sham group. Additionally, we hypothesized these improvements in the active tDCS group to be sustained for up to 1 month after the intervention.

## Methods

### Study design and setting

The study was a prospective, double-blind, randomized, sham-controlled trial. All outcome assessments were collected at the Faculty of Physical Therapy, Mahidol University, Thailand, while home-based intervention sessions were performed by participants at their residences and were supervised by a researcher via video conference. Ethical approval was obtained from the Mahidol University Central Institutional Review Board (MU-CIRB 2023/081.2305). The trial was registered prospectively on the ClinicalTrials.gov (NCT06079138).

### Study participants

**Fig. 1:**
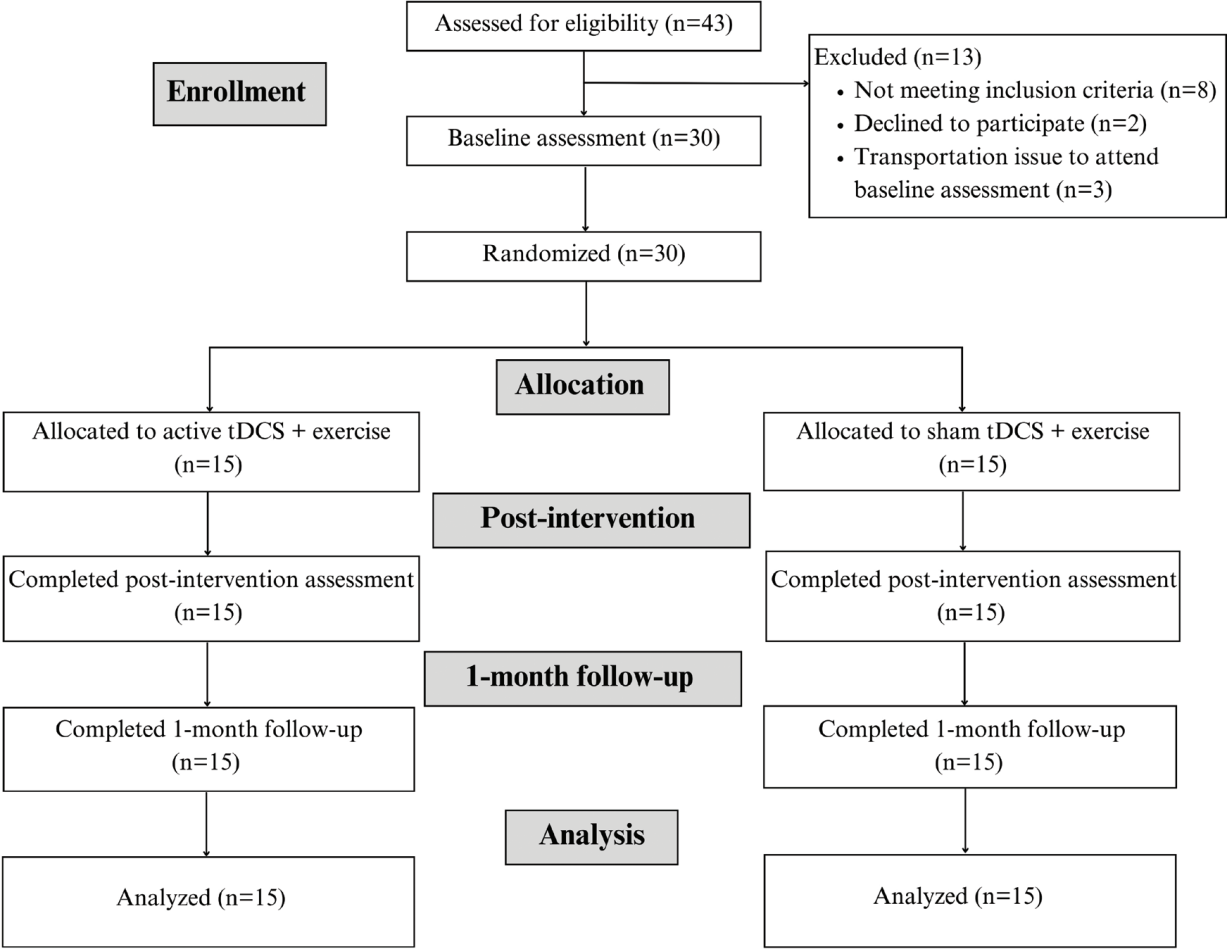
Study flow diagram.

Participants were recruited from discharged patients at the Sirindhorn National Medical Rehabilitation Institute (SNMRI) in Nonthaburi, Thailand and from online social-media platform communities for people with SCI during the period of data collection (July 2023–April 2024). Forty-three individuals with SCI were assessed for eligibility. Of these, 30 participants were enrolled in the study (*n* = 15 active tDCS; *n* = 15 sham tDCS). No participant withdrew from the study, and there were no dropouts (Fig. 1). The 30 participants were recruited based on the following inclusion criteria: (*i*) traumatic or non-traumatic SCI (American Spinal Injury Association Impairment Scale [AIS] A–D); (*ii*) aged between 18 and 70 years old; (*iii*) onset of injury between 1 and 30 months. Exclusion criteria included: (*i*) moderate-to-high musculoskeletal pain (numeric pain score > 4/10) limiting daily activities; (*ii*) receiving other non-invasive brain stimulation and alternative medicine such as transcranial magnetic stimulation (TMS), peripheral magnetic stimulation (PMS), or acupuncture; (*iii*) unstable clinical signs such as chest pain, resting heart rate >100 bpm, systolic blood pressure ≥180 and/or diastolic ≥ 100 mmHg; (*iv*) uncontrolled conditions such as hypertension or diabetes mellitus; (*v*) history of other neurological diseases; (*vi*) contraindications to tDCS use, including metal implantation, open scalp wounds, and epilepsy history.

### Randomization

Randomization occurred after participants were determined to be eligible for the study. They were randomly assigned to either the anodal or sham group using sealed envelopes marked “A” (anodal) or “B” (sham). Once the first participant was randomized, the next participant was considered based on a matched-pair design, accounting for 2 factors: (*i*) level of injury (tetraplegia or paraplegia) and (*ii*) onset (subacute <12 months or chronic ≥ 12 months). If no suitable match was found or a new pair was needed, randomization was repeated. tDCS was programmed according to group assignment and locked with a code to maintain blinding. The randomization process and tDCS programming were conducted by an independent researcher who was not involved in intervention delivery or assessments. Additionally, throughout the study, participants, outcome assessors, and physical therapists remained unaware of group assignments to reduce the potential for bias and ensure the integrity of the results.

### Intervention

**Fig. 2:**
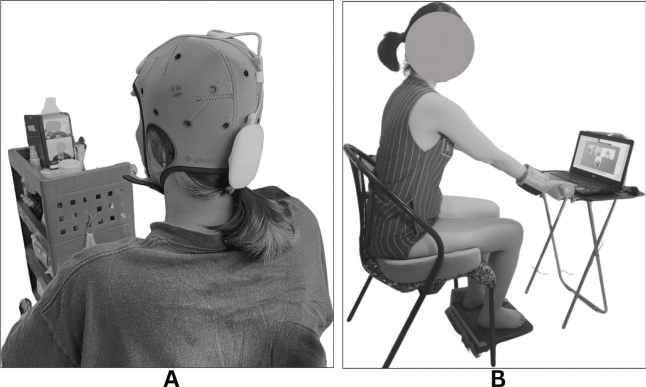
Intervention. (A) Participant applied tDCS at home and (B) participant was performing the upper limb exercise. The tDCS and exercise sessions were under supervision via video conference.

Tele-rehabilitation sessions were conducted via video conference for 12 sessions (3 times a week for 4 weeks). Each session included 20 min of active- or sham-tDCS, followed by a 1-hour exercise programme. Both groups received the same exercise programme (Fig. 2).

Participants received a tDCS set for home use and were trained along with their caregivers. The current stimulator was delivered with a battery-driven constant current stimulator (Ybrain, MINDD STIM, Republic of Korea). The stimulator used rectangular saline-soaked sponge-pad electrodes (35 cm²). The anodal electrode was placed over the vertex (Cz) and the cathodal over the supraorbital region (Fp1 or Fp2), contralateral to the more affected limb (18). The motor scores, obtained from the International Standards for Neurological Classification of Spinal Cord Injury (ISNCSCI) assessment, were used to determine the more affected limb, with the limb receiving lower motor scores identified as the more affected side. In cases of symmetrical lower limb weakness, the cathodal electrode was applied over the supraorbital region on the dominant side of the brain. Electrodes were fixed to a cap, which was fitted to each participant’s head. Participants placed the cap themselves or with caregiver assistance.

The active group received 2 mA active tDCS with a 30-sec ramp up and down. In the sham group, the current was delivered only for the first 30 s before being automatically terminated, while the electrodes remained on the participant’s head for 20 min, following a previously used protocol (18). Both groups heard a beeping sound during the 20-min stimulation. There was no change for tDCS setting throughout the 4-week protocol. Participants reported their feelings during and after stimulation, and adverse effects were recorded. Once the participants returned the tDCS, we were able to review the recorded stimulation history to verify the success of each session.

The exercise programme consisted of arm stretching, upper and lower limb resisted exercises, functional balance training, and functional training for wheelchair or bed transfers. However, the exercises were adjusted based on the participants’ abilities. A progressive resistance exercise programme was based on 1 repetition maximum (1RM). For the first 2 weeks, participants performed 3 sets of 10 repetitions at 75% of their 1RM. A new 1RM was assessed at the beginning of the third week, and exercise intensity was adjusted accordingly.

### Data collection

Baseline, post-intervention (a day after 12-session), and 1-month follow-up assessment were conducted by an assessor who was blinded to group allocation.

### Primary outcomes

The assessor, a physical therapist with more than 1 year of experience in the neurological field, completed 4-h informal training in the lab. This training included self-study using the booklet of the International Standards for Neurological Classification of Spinal Cord Injury (ISNCSCI) (19), practice cases from the classification workbook (20), and case discussion with experts. The same assessor assessed outcomes at baseline, post-intervention, and follow-up assessments.

The motor and sensory scores using the ISNCSCI assessment were the primary outcomes. The motor score of 5 key muscle functions of the upper extremities (UEMS) and 5 key muscle functions of the lower extremities (LEMS) was the primary outcome. The scores assess isotonic muscle contractions through key muscles linked to their corresponding dermatomes. It was graded on a scale of 0 to 5, with 5 representing normal motor function and 0 representing total paralysis, totalling 50 scores for each upper and lower extremities. For sensory scores, it assessed the ability to perceive light touch (light touch scores) and pinprick (pinprick scores) at the dermatomes from C2 to S5 (28 sensory key points). It was graded on a scale of 0 to 2, with 2 representing intact sensation and 0 representing absence, totalling 112 scores for each light touch and pinprick. Higher motor and sensory scores indicate better motor and sensory function (21).

### Secondary outcomes

*Daily activity and functional performance.* The spinal cord independence measure III (SCIM-III) assessed each participant’s performance in activities of daily living and mobility. Two domains of SCIM-III, self-care (6 items) and mobility (9 items) were used. The total score of self-care ranged from 0 to 20 and mobility scores ranging from 0 to 40, with higher scores indicating greater performance in activities of daily living and mobility (22).

*Transfer performance.* The transfer assessment instrument version 4.0 (TAI) assessed transfer skills by wheelchair. Each item in the TAI is scored “yes” (1 point), “no” (0 points), or “not applicable” (item not included in scoring), resulting in a minimum score of 0 and maximum score of 10. A higher TAI score indicates better transfer skills (23).

*Quality of life.* The 26-item World Health Organization Quality of Life Brief–Thai (WHOQOL-BREF–Thai) assessed quality of life in 4 domain scores: physical, social, and environmental, with 2 additional individually scored items concerning an individual’s overall perception of quality of life and health. The total scores range from 0 to 130 and a higher score indicates better quality of life (24).

*Spasticity outcomes.* Spasticity can be measured by electrophysiological and clinical assessments. The soleus H-reflex is an equivalent of the monosynaptic stretch reflex, and commonly used to assess spinal motoneuron excitability (25). Participants were positioned prone with the ankle in a neutral position (26). The soleus H-reflex was evoked through electrical stimulation of the tibial nerve at the popliteal fossa using a bipolar probe electrode (Medelec Synergy EMG and EP systems, VIASYS Healthcare, UK). For muscle activity recording, 2 disposable surface Abingdon, UK were placed over the motor point of the soleus muscle (lower 1/3 of the lower leg) and a ground electrode was placed over the lateral malleolus. The maximum H-reflex amplitude (H_max_), the maximum M response amplitude (M_max_), and H-reflex latency were recorded. Higher H_max_/M_max_amplitude ratio (H_max_/M_max_ ratio) and decrease in H-reflex latency indicates a high degree of muscle spasticity (27, 28).

The modified-Modified Ashworth Score (m-MAS) was used as the clinical outcomes to assess muscle tone of the knee extensors and ankle plantar flexor. The m-MAS was graded on a 6-point scale, range from 0 to 5 (29). Higher m-MAS scores indicate increased level of spasticity.

*Upper limb muscle strength test.* The isometric muscle strength of 8 muscle groups was measured in newtons using a hand-held dynamometer (Lafayette Electronic Hand-held Dynamometer; Lafayette Instrument, Lafayette, IN, USA): shoulder flexion, shoulder abduction, shoulder extension, shoulder adduction, elbow flexion, wrist extension, elbow extension, and wrist flexion on both sides. Participants were asked to exert maximum force against the dynamometer for 5 s (30). The average peak force (in newtons) of 2 repetitions for each muscle was analysed. A higher peak force score indicates better muscle strength. This measurement reflects maximal isometric contractions and quantifies muscle strength, allowing us to observe changes consistent with the outcomes of our exercise programme.

### Statistical analysis

As no existing evidence was available on the effects of home-based tDCS combined with exercise in the SCI population, we based our sample size calculation on a repeated 2-way analysis of variance (ANOVA), aligned with our study design. We determined the input parameters for a between-group comparison using an effect size off = 0.25 (medium), as recommended for tDCS studies (31), with α = 0.05, power = 0.8, and 3 time points. Results showed that a sample size of at least 28 (14 participants per group) was adequate to attain reliable effects. Therefore, 30 participants were enrolled, and data from 30 participants were used for statistical analysis.

The χ^2^ test, Fisher’s exact test, independent samples *t*-tests, or Mann–Whitney *U* test were used to compare baseline characteristics and clinical outcomes between groups. For primary and secondary outcomes data, change scores from individual baseline data were used for analysis and the calculated formulas were as follows: (1) At post = post-intervention – baseline, (2) At follow-up = 1-month follow-up – baseline. The normality of the distribution was verified using the Shapiro–Wilk test. Between-group comparisons (group effect), within-group comparisons (time effect), and interaction effect (group x time) were performed using 2-way mixed analysis of variance (ANOVA) followed by Bonferroni post-hoc tests if data were normally distributed data. For non-normally distributed data, Mann–Whitney *U* tests were used to analyse between-group differences at each time point, while within-group differences were analysed using the Friedman test. The significance level was set at *p* < 0.05 (two-sided). To control the Type I error rate, multiple post-hoc comparisons using the Bonferroni correction were performed if any significant main effect or interaction effect was observed. Bonferroni’s correction (*p* = 0.05/4, thus *p* = 0.0125) was applied for multiple comparisons, because there are 4 comparisons (2 time points × 2 groups).

## Results

Participant baseline characteristics and clinical outcomes are presented in Table I. There were differences in the absolute numbers of age, gender, aetiology, severity, onset of injury, or ISNCSCI scores between groups; however, no statistical differences were found. For the H_max_/M_max_ ratio and H-reflex latency, we obtained data from only 10 participants in the active group, and 9 participants in the sham group due to absence of H-reflex in those participants. All participants reported being able to apply the tDCS at home. Eight out of 30 participants (27%) required assistance to set up the home-use tDCS kit, such as wearing the cap, placing the sponge-pad electrode, and turning on the tDCS due to upper limb weakness. For tDCS-related adverse events, only mild cutaneous sensation was noted such as mild tingling (active, 93%; sham 93%), mild itching (active, 87%; sham, 67%), and mild burning sensation (active, 53%; sham, 60%). These adverse effects mostly occurred during the first few minutes after stimulation and disappeared after stimulation ending. None of the participants received functional and transfer training via tele-rehabilitation, as they were unable to perform it independently.

**Table I:** Baseline characteristics, clinical outcomes, and statistical analysis

Variables	Active tDCS group (*n*=15)	Sham tDCS group (*n*=15)	*p*-value
Age, years, mean (SD)	42.7 (11.6)	41.2 (12.8)	0.400^a^
Gender, female/male, *n*	5/10	6/9	0.144^b^
Aetiology, *n*			0.543^b^
Traumatic	14	13	
Non-traumatic	1	2	
Severity of injury, *n*			0.940^b^
American Spinal Injury Association Impairment Scale A	4	5	
American Spinal Injury Association Impairment Scale B	4	3	
American Spinal Injury Association Impairment Scale C	3	4	
American Spinal Injury Association Impairment Scale D	4	3	
Level of injury, *n*			0.715^b^
C4–C7	8	6	
T4–T12	4	7	
L1–L5	3	2	
Onset of injury, months, mean (SD)	12.9 (9.1)	16.5 (7.6)	0.419^a^
Range of onset of injury, *n*			
0–3 months	1	1	0.135^b^
4–6 months	4	1	0.392^b^
7–12 months	5	3	0.449^b^
>12 months	5	10	0.589^b^
ISNCSCI, mean (SD)			
UEMS	40.9 (10.9)	42.1 (9.8)	0.740^a^
LEMS	10.3 (10.8)	8.4 (13.4)	0.358^c^
Light touch	75.7 (21.9)	69.9 (25.1)	0.395^c^
Pinprick	75.6 (23.0)	69.9 (26.1)	0.533^a^

a Data were analysed by independent *t*-test.

b Data were analysed by χ^2^ test.

c Data were analysed by Mann–Whitney *U* test.

tDCS: transcranial direct current stimulation; ISNCSCI: International Standards for Neurological Classification of Spinal Cord Injury, which includes assessments of Upper Extremity Motor Score (UEMS), Lower Extremity Motor Score (LEMS), light touch, and pinprick.

### Primary outcomes

**Fig. 3:**
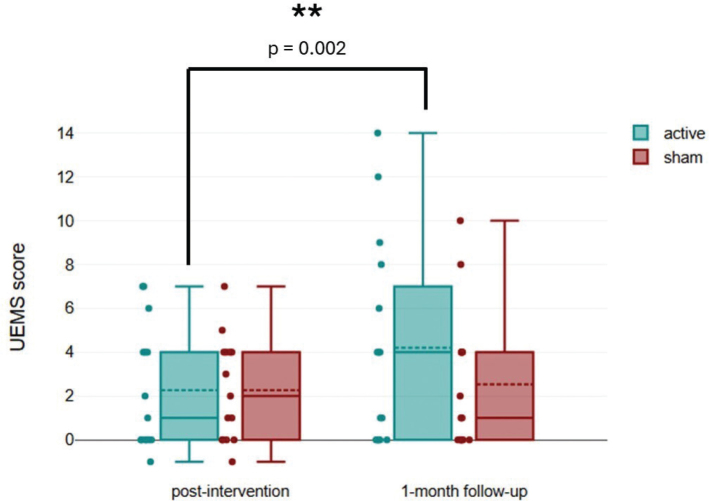
Comparison of change score from baseline between groups for Upper Extremity Motor Score (UEMS).

The summarized results of primary outcomes are presented in Table II. For UEMS, the active group showed an increase of 2.3 points 95% CI (0.71–3.83) at post-intervention and 4.2 points (1.61–6.79) at 1-month follow-up, while the sham group showed an increase of 2.3 points (0.97–3.56) at post-intervention and 2.5 points (0.80–4.27) at 1-month follow-up. A 2-way mixed ANOVA revealed significant interaction effects between group and time (F_(1,18)_= 4.49, *p* = 0.043) and main effects of time (F_(1,18)_ = 7.82, *p =* 0.009). Bonferroni *post-hoc* analysis showed a significant improvement only within the active group (*p =* 0.002). This indicates that active tDCS led to greater improvement of UEMS than sham group over time (Table II, Fig. 3). There were no significant differences between the active and sham groups for the LEMS and sensory scores (light touch and pinprick scores) at post-intervention and 1-month follow-up (Table II).

**Table II:** Summarized results of ISNCSCI scores, SCIM-II, TAI score, H-reflex, m-MAS, and *p*-value from statistical analysis

Outcomes	Group	Change score from baseline				*p*-value					Group x time effect

		At Post	95%CI	At 1M	95%CI	Time effect (within-group comparisons)		Group effect (between-group comparisons)			

						Overall	At post vs 1M	Overall	At post	At 1M	
Primary outcome measures											
UEMS^a^	Active	2.3 (2.8)	0.71–3.83	4.2 (4.7)	1.61–6.79	0.009**	0.002**	0.479	1	0.183	0.043*
	Sham	2.3 (2.3)	0.97–3.56	2.5 (3.1)	0.80–4.27		0.635				
LEMS^b^	Active	0.0 (0.0, 4.0)	–0.75–3.02	0.0 (–1.0, 3.0)	–1.39–4.19	–	0.888	–	0.965	0.947	–
	Sham	0.0 (0.0, 1.0)	–0.20–2.33	0.0 (0.0, 1.0)	–0.53–1.86		0.336				
Light touch^b^	Active	0.0 (–3.0, 6.0)	–4.08–6.48	0.0 (–3.0, 8.0)	–2.14–6.54	–	0.937	–	0.429	0.359	–
	Sham	3.0 (0.0, 9.0)	0.21–8.06	5.0 (–1.0,10.0)	0.88–9.25		0.653				
Pinprick^a^	Active	3.5 (10.2)	–2.11–9.17	3.7 (11.0)	–2.40–9.74	0.565	–	0.976	–	–	0.69
	Sham	3.3 (7.8)	–1.01–7.67	4.1 (7.6)	–0.13–8.26		–				
Secondary outcome measures											
SCIM self-care domain^b^	Active	0.0 (–1.0, 1.0)	–1.33–1.46	1.0 (0.0, 1.0)	–0.30–2.03	–	0.117	–	0.129	0.632	–
	Sham	0.0 (0.0, 2.0)	–0.01–2.41	0.0 (0.0, 2.0)	–0.07–2.07		0.496				
SCIM mobility domain^a^	Active	0.7 (2.2)	–0.57–1.90	0.7 (1.7)	–0.21–1.68	0.669	–	0.19	–	–	0.943
	Sham	1.7 (3.4)	–0.16–3.62	2.3 (3.9)	0.16–4.51		–				
TAI^b^	Active	0.0 (0.0, 2.1)	–0.15–2.71	0.1 (0.0, 1.6)	–0.24–1.89	–	0.767	–	0.85	0.834	–
	Sham	0.0 (0.0, 0.8)	–0.41–1.80	0.2 (–0.2, 0.9)	–0.32–2.57		0.625				
WHOQOL^a^	Active	1.3 (11.4)	–5.02–7.56	2.3 (9.4)	–2.93–7.47	0.423	–	0.715	–	–	0.942
	Sham	0.1 (6.1)	–3.30–3.43	1.3 (8.2)	–3.52–5.80		–				
Contralateral (contralateral limb to the stimulation brain)											
H-reflex latency^b^	Active	–1.38 (–3.72, 0.28)	–5.91–0.67	–0.07 (–2.42, 1.25)	–5.82–4.66	–	0.374	–	0.369	0.369	–
	Sham	–0.35 (–2.37, 0.30)	–2.77–0.54	–0.55 (–4.22, 0.15)	–3.47–0.22		0.407				
H_max_/M_max_ ratio^b^	Active	0.07 (–0.14, 0.13)	–0.16–2.57	0.07 (–0.07, 0.31)	–0.50–0.29	–	0.386	–	0.461	0.838	–
	Sham	–0.01 (–0.13, 0.08)	–0.15–0.17	0.08 (–0.08, 0.26)	–0.07–0.22		0.161				
m-MAS of ankle plantar flexors^b^	Active	0.0 (0.0, 0.0)	–0.88–0.34	0.0 (–1.0, 0.0)	–1.21–0.55	–	0.589	–	0.17	0.046	–
	Sham	0.0 (0.0, 0.0)	–0.22–1.02	(0.0 (0.0, 1.0)	–0.48–1.68		0.498				
m-MAS of knee extensors^b^	Active	0.0 (0.0, 0.0)	–0.15–0.42	(0.0 (0.0, 1.0)	–0.47–0.60	–	0.518	–	1	0.536	–
	Sham	0.0 (0.0, 0.0)	–0.27–0.80	0.0 (0.0, 0.0)	–0.11–0.84		0.414				
Ipsilateral (ipsilateral limb to the stimulation brain)											
H-reflex latency^b^	Active	–0.12 (–0.86, 0.91)	–8.53–14.52	0.35 (–2.15, 3.52)	–5.02–6.37	–	0.959	–	0.806	0.327	–
	Sham	0.05 (–3.47, 0.77)	–2.82–0.76	–0.30 (–2.60, 0.42)	–2.29–0.36		0.767				
H_max_/M_max_ ratio^b^	Active	0.02 (–0.08, 0.17)	–0.12–0.22	–0.03 (–0.07, 0.06)	–0.15–0.27	–	0.721	–	0.967	0.27	–
	Sham	0.02 (–0.10, 0.21)	–0.08–0.18	0.19 (–0.14, 1.32)	–0.45–2.06		0.401				
m-MAS of ankle plantar flexors^b^	Active	0.0 (0.0, 0.0)	–0.60–0.33	0.0 (0.0, 0.0)	–1.06–0.53	–	0.465	–	0.057	0.614	–
	Sham	0.0 (0.0, 2.0)	0.05–1.28	0.0 (0.0, 1.0)	–0.81–1.47		0.246				
m–MAS of knee extensors^b^	Active	0.0 (0.0, 0.0)	–0.18–0.71	(0.0 (0.0, 1.0)	–0.56–0.82	–	1	–	0.587	0.535	–
	Sham	0.0 (0.0, 0.0)	–0.09–1.16	(0.0 (0.0, 1.0)	0.03–1.04		0.785				

a Data presented as mean (SD),

b Data presented as median (Q1, Q3).

ISNCSCI: International Standards for Neurological Classification of Spinal Cord Injury, which includes assessments of UEMS, LEMS, light touch, and pinprick; UEMS: Upper Extremity Motor Score; LEMS: Lower Extremity Motor Score; SCIM-III: Spinal Cord Independence Measure III; TAI: Transfer Assessment Instrument; WHOQOL-BREF–Thai: The World Health Organization Quality of Life Brief–Thai; H_max_/M_max_ ratio: ratio between maximum H-reflex response amplitude and maximum M-wave response amplitude; m-MAS: *modified*-Modified Ashworth score; Higher scores indicate better performance for UEMS, LEMS, light touch, pinprick; SCIM self-care domain; SCIM mobility domain; TAI, WHOQOL.

Overall significance *p*-value <0.05, *p*-value after Bonferroni correction for pairwise comparison *p*<0.0125.

* *p*<0.05;

** *p*<0.01.

### Secondary outcomes

The summarized results for secondary outcomes are presented in Table II. There were no significant within-group and between-group differences for secondary outcomes including H-reflex, upper limb muscle strength, m-MAS, SCIM-III scores, TAI score, and quality of life (Table II and Table III) at post-intervention and 1-month follow-up. Additionally, raw data of all outcomes are presented in Tables SI and SII.

**Table III:** Summarized results of muscle strength and statistical analysis

Outcomes	Group	Change score from baseline				*p*-value					Group x time effect

		At post	95% CI	At 1M	95% CI	Time effect (within-group comparisons)		Group effect (between-group comparisons)			

						Overall	At 1M vs post	Overall	At post	At 1M	
Contralateral (contralateral limb to the stimulation brain)											
Shoulder flexors^a^	Active	28.22 (33.28)	–0.18–29.65	27.68 (31.68)	–5.11–36.04	0.255	–	0.453	–	–	0.216
	Sham	29.61 (21.40)	8.73–33.24	42.04 (37.69)	14.03–52.05		–				
Shoulder abductors^a^	Active	18.43 (20.96)	3.63–30.45	21.25 (29.13)	7.32–36.15	0.294	–	0.144	–	–	0.644
	Sham	32.15 (35.54)	11.43–38.73	39.33 (38.13)	16.26–43.54		–				
Shoulder extensors^a^	Active	11.41 (23.09)	–0.71–24.02	16.18 (27.90)	3.33–33.75	0.925	–	0.389	–	–	0.238
	Sham	22.81 (21.36)	11.01–41.84	18.74 (23.23)	17.98–36.70		–				
Shoulder adductors^b^	Active	23.45 (2.90, 30.00)	7.71–36.36	17.90 (1.40, 35.00)	20.47–45.42	–	0.733	–	0.576	0.093	–
	Sham	22.55 (5.80, 45.45)	19.99–38.26	29.25 (13.85, 68.00)	19.90–50.69		0.112				
Elbow flexors^a^	Active	23.94 (24.84)	7.45–46.28	17.94 (34.30)	7.60–42.34	0.790	–	0.296	–	–	0.355
	Sham	30.60 (35.88)	11.89–46.99	33.93 (32.49)	15.90–46.79		–				
Wrist extensors^a^	Active	10.69 (22.98)	–2.14–18.30	16.16 (21.77)	7.74–30.64	0.034	–	0.107	–	–	0.379
	Sham	21.79 (25.85)	7.76–32.70	34.58 (34.44)	14.33–43.48		–				
Elbow extensors^b^	Active	1.55 (0.00, 14.45)	–6.06–25.10	6.85 (0.15, 14.85)	1.37–26.61	–	0.173	–	0.885	0.494	–
	Sham	4.40 (–3.30, 19.55)	–3.61–19.23	8.65 (1.15, 38.45)	1.62–36.52		0.140				
Wrist flexors^a^	Active	10.79 (18.40)	5.31–33.92	15.07 (19.48)	5.08–32.13	0.062	–	0.063	–	–	0.448
	Sham	21.21 (20.58)	5.57–33.97	31.13 (25.83)	–0.63–34.61		–				
Ipsilateral (ipsilateral limb to the stimulation brain)											
Shoulder flexors^a^	Active	14.82 (26.78)	9.79–46.65	15.46 (37.15)	10.14–45.23	0.188	–	0.253	–	–	0.236
	Sham	20.98 (22.12)	17.75–41.46	33.04 (34.33)	21.16–62.91		–				
Shoulder abductors^a^	Active	17.04 (24.21)	6.82–30.04	21.74 (26.02)	5.11–37.38	0.195	–	0.340	–	–	0.986
	Sham	25.08 (24.64)	12.47–51.83	29.90 (24.63)	18.21–60.45		–				
Shoulder extensors^a^	Active	11.66 (22.32)	–1.37–24.20	18.54 (27.46)	0.72–31.63	0.348	–	0.140	–	–	0.473
	Sham	26.42 (27.83)	10.97–34.64	27.35 (16.90)	5.88–31.61		–				
Shoulder adductors^a^	Active	22.04 (25.86)	10.63–31.38	32.94 (22.53)	6.54–32.29	0.082	–	0.517	–	–	0.620
	Sham	29.13 (16.50)	13.71–46.10	35.30 (27.80)	19.41–74.11		–				
Elbow flexors^a^	Active	26.87 (35.05)	10.17–37.70	24.98 (31.36)	–1.06–36.94	0.999	–	0.669	–	–	0.714
	Sham	29.44 (31.69)	10.73–50.48	31.34 (27.88)	15.93–51.92		–				
Wrist extensors^a^	Active	8.08 (18.45)	–2.04–23.42	19.20 (20.67)	4.10–28.22	0.009	–	0.146	–	–	0.730
	Sham	20.23 (22.52)	7.47–36.11	28.90 (26.32)	15.50–53.65		–				
Elbow extensors^a^	Active	9.52 (28.13)	–16.08–14.51	13.99 (22.79)	2.70–17.27	0.100	–	0.840	–	–	0.468
	Sham	7.81 (20.61)	–0.41–15.47	19.09 (31.48)	3.35–43.74		–				
Wrist flexors^a^	Active	19.62 (25.82)	0.59–20.98	18.61 (24.41)	4.28–25.86	0.701	–	0.933	–	–	0.857
	Sham	19.77 (25.63)	9.81–32.61	16.99 (31.81)	16.82–45.44		–				

Higher scores indicate better muscle strength performance.

a Data presented as mean (SD),

b Data presented as median (Q1, Q3).

Overall significance *p*-value <0.05, p-value after Bonferroni correction for pairwise comparison *p*<0.0125.

## DISCUSSION

This study found that 12 sessions of home-based active tDCS combined with a tele-exercise programme significantly improved UEMS in the active tDCS group over the sham group. No significant between-group differences were found for LEMS, sensory scores, or any secondary outcomes, including spasticity outcomes, the lower limb muscle strength, functional and transfer performance, and quality of life.

### tDCS application

Our study used unilateral tDCS, with the anodal electrode positioned over the vertex (Cz), and the centre of the large side of the electrode (7 cm) was in the horizontal plane covering the primary motor cortex (M1) of both hemispheres. The cathodal electrode was positioned over the supraorbital region on the contralateral side to the limb with greater motor impairment. This configuration aims to facilitate the M1 of both hemispheres with more focus on the hemisphere controlling the more impaired side. However, it should be noted that the anodal electrode was positioned closer to the lower limb M1, which is located next to the vertex compared with that of the upper limb. Nevertheless, the non-focal nature of conventional tDCS leads to activation of a wide area, not just underneath the electrode (32). The intensity used here was 2 mA, as it has been reported that anodal tDCS at 2 mA could induce change in the excitability of the lower leg motor area (33), and may stimulate spinal pathways in the SCI population (8).

### Effect on motor and sensory function

The motor score (UEMS) reached the minimally detectable change (MDC) for SCI in both groups (> 0.29 points) (34). For minimal important difference (MID) this can be used to gauge clinical significance. It has been reported that a clinically meaningful change in SCI requires approximately 3 points for UEMS, 3.5 for LEMS, and 5.5 for sensory (35). Based on our result, only the UEMS changes observed at 1 month follow-up in the active group (4.2 points) met this threshold. This may be attributed to the combination of anodal tDCS and exercise. Our tele-exercise programme consisted of 12 sessions (3 sessions per week for 4 weeks), a shorter duration than a typical resistance training programme that significantly increases muscle strength in individuals with SCI, which usually lasts 6 to 12 weeks with 2–5 sessions/week (36). However, our exercise programme was tailored to each participant’s ability, and all participants (100%) were able to complete the upper limb exercise. This result agreed with several tDCS studies that have reported positive effects of active tDCS combined with upper limb motor training in individuals with SCI at immediately post-intervention (17, 37–40), with long-lasting improvement (17, 38). However, it should be noted the improvement of UEMS in the active group was more dominant at the follow-up, not immediately. This may be due to several reasons with a delayed effect of tDCS being a possible explanation. A systematic review reported in healthy individuals that anodal tDCS over the cerebellum for a single-session enhanced motor skill learning in the short (<24 h) and long-term (>24 h) post-intervention, while no effect of motor learning immediately after or during stimulation was observed (41). Similarly, a delayed peak effect of tDCS on upper limb movement was reported 24 h after a single session of tDCS over M1 in a stroke population (42). In people with a major depressive disorder, a peak effect on depressive outcome was not observed immediately after multiple sessions of tDCS (i.e., 12 sessions). Significant improvement emerged 4 weeks post-intervention and reached its maximum at 6 weeks post-intervention (43). The after-effect of tDCS has been observed to involve neuroplastic changes such as long-term potentiation (LTP) via NMDA receptors (44), as well as a non-synaptic mechanism (45). These processes are essential for motor learning (46). In addition, the effect of tDCS on motor learning appears to depend on the interaction between tDCS parameters and motor task characteristics, including the number of sessions and the amount of practice (47). The delayed effect may be due to tDCS-induced LTP-like plasticity, which strengthens task-relevant neuronal connections over long periods after stimulation.

tDCS application seems to reach lower limb M1, but no significant improvement was found for lower limb function. Previous studies demonstrated that tDCS with robotic and overground gait training could improve lower limb motor performance in individuals with incomplete SCI (18, 47–49). These studies showed positive effects following a specific motor task with tDCS application. A systematic review reported that the tDCS effect was dependent on motor exercise characteristics together with specificities of tDCS application (47). This may explain the lack of improvement in lower limb motor performance in this study, as about 50% of participants did not receive lower limb exercise due to their high level of lower limb impairment. Eleven participants (active *n*=6, sham *n*=5) could perform lower limb exercise with some assistance. Only 4 participants (active *n*=2, sham *n*=2) could perform the exercises independently, but they were unable to progress with the exercise due to their impairment.

No significant changes were observed in any group for sensory scores. Mean changes in both groups remained below the MDC levels for both light touch (<12.96 points) and pinprick scores (<7.8 points) (34). Our results disagreed with a previous tDCS study. Murray et al. demonstrated that 3 sessions of 1 or 2 mA of anodal tDCS over the M1 of extensor carpi radialis significantly reduced the sensory perceptual threshold of the radial nerve in individuals with incomplete SCI (8) However, it should be noted that the sensory threshold in the study by Murray et al. was evaluated by peripheral electrical stimulation, not clinical outcomes. To date, there is limited evidence regarding the effects of tDCS on sensory recovery in the SCI population.

### Effect on spasticity

The effect of tDCS on spasticity in individuals with neurological disorders is controversial, and the optimal dosage treatment for spasticity remains unclear (50). In incomplete SCI, anodal tDCS combined with patterned electrical stimulation on spinal inhibitory interneurons was shown to increase reciprocal inhibition and presynaptic inhibition of the soleus H-reflex and increase ankle movement after stimulation (40). It was proposed that improvement of ankle movement could be due to a reduction of spasticity in the ankle plantar flexor (40). Anodal tDCS has the potential to reduce spasticity in multiple sclerosis patients, as shown by significantly reducing the H_max_/M_max_ ratio with no change of MAS (51). MAS was suggested to be an insensitive measure for spasticity compared with the H_max_/M_max_ ratio (52). However, we did not find any significant changes in either the m-MAS or the H_max_/M_max_ ratio in the present study. It should be noted that H-reflex was elicited in only 63% of participants (active *n* =10, sham *n* =9), making it difficult to interpret or generalize the spasticity-related results.

### Effect on functional and transfer function

Minimal and insignificant improvements were observed in the SCIM-III and TAI for both groups, likely because none of the participants received functional and transfer training. This was due to their need for assistance, lack of confidence, and environmental challenges at home such as limited space, unsafe or substandard beds, chairs, or wheelchairs, and mismatched heights between their wheelchairs and beds. Our finding suggests that strength training alone is insufficient to improve functional and transfer ability in individuals with SCI.

### Home-based intervention

tDCS combined with tele-rehabilitation presents an innovative approach to treat individuals with SCI. Overall, participants in both groups completed 100% of 12 sessions, suggesting good adherence to home-based intervention. This intervention ensures continuity of care by enabling patients to receive ongoing rehabilitation in the comfort of their own homes, thereby improving accessibility and minimizing barriers to treatment. Moreover, this approach offers substantial time and cost efficiencies, addressing many of the challenges faced by SCI patients and healthcare providers alike.

### Limitations and suggestions

First, this study includes a diverse range of levels and severity of spinal cord injury (see Table I). We included 16 individuals with motor complete SCI (AIS A or B) (*n* =8 in the active group, *n* =8 in the sham), and 14 individuals with motor incomplete SCI (AIS C or D) (*n* =7 in the active group, *n* =7 in the sham). Although participant characteristics were similar between groups, level and severity of injury can influence recovery (53). As individuals with motor complete injuries lack voluntary movement below the level of injury, their capacity for motor recovery is limited. This may have influenced our findings, particularly for lower limb outcome. Our sample size was too small to conduct a subgroup analysis to assess the effects on motor complete and incomplete SCI. Second, although we used a medium effect size for sample size calculation, as recommended by Mitra et al. (31) for tDCS studies, our total sample size of 30 still falls within the range (22.2±24.9 subjects) identified as potentially underpowered (31). Future studies with a larger sample size and specific level and severity profiles are recommended. Third, while we applied the Bonferroni correction to control for multiple comparisons, it may not fully account for the multiple testing performed. Given that the primary outcome consisted of 4 subscales and multiple comparisons, the risk of Type I error remains a consideration. Fourth, tele-exercise may be less effective for individuals with severe complications or those who require assistance, and functional and transfer exercises may not suit tele-supervision. Fifth, 37% of participants exhibited an absence of H-reflex, which limited the interpretation of spinal motoneuron excitability, and spasticity-related results. Sixth, due to ethical considerations, participants were permitted to undertake continuous rehabilitation during follow-up. Eighteen participants (active *n*=10, sham *n*=8) continued rehabilitation, and 12 participants (active *n*=5, sham *n*=7) maintained their home exercise. This might have affected follow-up results; however, both groups received the same type and amount of training as recorded in their logbooks. Lastly, since individuals with motor complete injuries have minimal potential for motor recovery, selecting a primary outcome that is sensitive to changes across different severity levels is important. Future studies may consider alternative primary clinical outcomes, such as the Functional Independence Measure (FIM) or neurophysiological outcome to better capture the effects of intervention in individuals with motor complete SCI.

### Clinical implication

This is the first study to explore the effects of home-based tDCS combined with exercise through tele-rehabilitation in individuals with SCI. Results indicate that this approach is feasible, safe, and well-adhered to. However, there are clinical feasibility concerns, including increased costs for the tDCS device, the electronic devices with internet access (e.g., smart phone, tablet, computer), and the additional time needed for tDCS application, although it is possible to administer tDCS concurrently with exercise (54). For those with limited upper limb function, home-based tDCS may require caregiver assistance. Functional and transfer training may not be suitable for home-based exercise as it requires assistance. This combined approach is particularly suited for outpatients on waiting lists or those lacking access to rehabilitation services. However, it should be noted that with the intervention used in the present study, only the UEMS score, which evaluates upper limb motor function, reached the clinically meaningful threshold. Caution should be taken when considering its clinical implementation. Further research is needed to determine the optimal dosage for other outcomes, such as overall motor function, sensory function, and related functional abilities.

### Conclusion

The effects of 12 sessions of anodal tDCS combined with tele-supervised exercise were limited to improved upper limb motor recovery, with positive after-effects at 1 month post-intervention. No differences between groups were observed for sensory impairment or secondary outcomes including upper limb muscle strength, spasticity, functional and transfer performance, and quality of life. However, given the multiple comparisons performed, statistical limitation analysis should be acknowledged.

Although this study demonstrated that this combined intervention is a feasible, safe, and well-adhered to approach for home-based intervention in individuals with SCI, some limitations should be noted. Some participants required assistance and were unable to perform lower limb, functional, and transfer training independently. Only upper limb exercises could be performed by all participants, suggesting that tele-rehabilitation may not be suitable for all types of training. Nevertheless, this study provides valuable insight into the potential of combining tDCS and exercise in a tele-rehabilitation programme for outpatients with SCI.

## Supplementary Material

TELE-REHABILITATION USING TRANSCRANIAL DIRECT CURRENT STIMULATION COMBINED WITH EXERCISE IN PEOPLE WITH SPINAL CORD INJURY: A RANDOMIZED CONTROLLED TRIAL
