# Supplementary material for: TELE-REHABILITATION USING TRANSCRANIAL DIRECT CURRENT STIMULATION COMBINED WITH EXERCISE IN PEOPLE WITH SPINAL CORD INJURY: A RANDOMIZED CONTROLLED TRIAL
Source: J Rehabil Med. 2025 May 7;57:42353. doi: 10.2340/jrm.v57.42353 (PMC12067987; doi:10.2340/jrm.v57.42353)
Supplement: TELE-REHABILITATION USING TRANSCRANIAL DIRECT CURRENT STIMULATION COMBINED WITH EXERCISE IN PEOPLE WITH SPINAL CORD INJURY: A RANDOMIZED CONTROLLED TRIAL [file JRM-57-42353-s1.pdf]

Supplementary material has been published as submitted. It has not been copyedited, or typeset by Journal of Rehabilitation Medicine

Table SI. The raw data of ISNCSCI scores, SCIM-III, TAI score, H-reflex, m-MAS

Outcome	Groups	Baseline	Post-intervention (Post)	1-month follow-up (1M)
**Primary outcome measures**				
UEMS[a]	Active	40.9 (10.9)	43.1 (8.9)	45.1 (7.6)
	Sham	42.1 (9.8)	44.4 (8.9)	44.6 (8.7)
LEMS[b]	Active	8.0 (0.0, 19.0)	9.0 (0.0, 18.0)	6.0 (0.0, 18.0)
	Sham	0.0 (0.0, 20.0)	0.0 (0.0, 21.0)	0.0 (0.0, 22.0)
Light touch[b]	Active	84.0 (55.0, 91.0)	83.0 (57.0, 94.0)	86.0 (56.0, 92.0)
	Sham	75.0 (50.0, 85.0)	74.0 (50.0, 101.0)	76.0 (60.0, 99.0)
Pinprick[a]	Active	75.6 (22.9)	79.1 (20.9)	79.3 (21.9)
	Sham	69.9 (26.1)	73.3 (23.7)	74.0 (23.9)
**Secondary outcome measures**				
SCIM self-care domain[b]	Active	17.0 (14.0, 19.0)	17.0 (13.0, 20.0)	18.0 (17.0, 19.0)
	Sham	18.0 (13.0, 20.0)	19.0 (15.0, 20.0)	19.0 (14.0, 19.0)
SCIM Mobility domain[a]	Active	16.1 (7.5)	16.8 (7.8)	16.9 (7.7)
	Sham	16.0 (9.2)	17.7 (7.8)	18.3 (7.7)
TAI[b]	Active	7.4 (0.0, 8.7)	8.1 (6.9, 8.7)	8.0 (6.9, 8.8)
	Sham	8.0 (6.4, 8.7)	8.2 (7.6, 8.7)	8.3 (7.2, 9.1)
WHOQOL[a]	Active	80.5 (9.6)	81.7 (16.3)	82.7 (12.2)
	Sham	81.4 (16.1)	81.5 (14.1)	82.7 (13.6)
Contralateral (contralateral limb to the stimulation brain)				
H-reflex latency[b]	Active	33.95 (32.05, 35.20)	31.81 (26.81, 33.95)	33.03 (30.52, 35.77)
	Sham	33.00 (31.10, 37.52)	32.95 (31.20, 34.80)	33.05 (29.52, 34.32)

H_{max}/M_{max} ratio[b]	Active	0.53 (0.19, 0.60)	0.52 (0.13, 0.68)	0.60 (0.32, 0.75)
	Sham	0.57 (0.29, 0.68)	0.59 (0.31, 0.66)	0.58 (0.49, 0.65)
m-MAS of ankle plantar flexors[b]	Active	3.5 (0.0, 4.0)	3.0 (0.0, 4.0)	3.0 (0.0, 3.5)
	Sham	4.0 (0.0, 4.0)	4.0 (1.0, 4.0)	4.0 (0.5, 3.0)
m-MAS of knee extensors[b]	Active	3.0 (0.0, 3.5)	3.0 (0.0, 4.0)	1.0 (0.0, 4.0)
	Sham	0.5 (0.0,4.0)	3.0 (0.0,4.0)	2.0 (0.0, 4.0)
Ipsilateral (ipsilateral limb to stimulation brain)				
H-reflex latency[b]	Active	33.00 (30.18, 35.53)	32.73 (29.80, 37.02)	33.32 (30.72, 36.16)
	Sham	32.85 (31.00, 35.77)	32.05 (30.95, 33.90)	32.15 (30.65, 34.32)
H_{max}/M_{max} ratio[b]	Active	0.54 (0.25, 0.82)	0.61 (0.44, 0.74)	0.62 (0.32, 0.86)
	Sham	0.56 (0.29, 0.76)	0.66 (0.50, 0.77)	0.67 (0.63, 1.58)
m-MAS of ankle plantar flexors[b]	Active	3.5 (0.0, 4.0)	4.0 (0.0, 4.0)	1.0 (0.0, 4.0)
	Sham	4.0 (1.0,4.0)	4.0 (2.3, 4.0)	4.0 (0.5,4.0)
m-MAS of knee extensors[b]	Active	3.0 (0.0, 3.0)	3.0 (0.0, 4.0)	3.0 (0.0, 4.0)
	Sham	0.5 (0.0, 4.0)	3.0 (0.0, 4.0)	2.0 (0.0, 4.0)

Note: ISNCSCI = the International Standards for Neurological Classification of Spinal Cord Injury, which includes assessments of UEMS, LEMS, light touch, and pinprick. UEMS = Upper Extremity Motor Score. LEMS = Lower Extremity Motor Score., SCIM-III = Spinal Cord Independence Measure III, TAI = Transfer Assessment Instrument, WHOQOL-BREF-Thai = The World Health Organization Quality of Life Brief – Thai, H_{max}/M_{max} ratio = Ratio between maximum H-reflex response amplitude and maximum M-wave response amplitude, m-MAS = *modified*-Modified Ashworth score. Higher scores indicate better performance for UEMS, LEMS, light touch, pinprick, SCIM self-care domain, SCIM Mobility Domain, TAI, WHOQOL. Lower m-MAS scores indicate increased level of spasticity. Lower H_{max}/M_{max} ratio and longer H-reflex latency indicate a lower degree of muscle spasticity.

[a]Data was presented as mean (SD), [b]Data was presented as median (Q1, Q3).

Table SII. The raw data of muscle strength

Outcomes	Groups	Baseline	Post-intervention (Post)	1-month follow-up (1M)
Contralateral (contralateral limb to the stimulation brain)				
Shoulder flexors[a]	Active	127.04 (42.19)	155.26 (44.34)	154.72 (46.75)
	Sham	126.36 (42.24)	155.97 (46.21)	168.40 (55.18)
Shoulder abductors[a]	Active	114.49 (40.96)	132.91 (43.73)	135.73 (49.40)
	Sham	116.76 (36.25)	148.91 (47.13)	156.09 (42.82)
Shoulder extensors[a]	Active	112.57 (45.86)	123.98 (45.69)	128.74 (47.18)
	Sham	112.23 (23.12)	134.04 (31.90)	129.98 (34.38)
Shoulder adductors[b]	Active	137.70 (112.00, 194.30)	170.05 (133.55, 206.75)	167.90 (118.85, 214.05)
	Sham	143.25 (109.30, 167.95)	163.75 (145.85, 203.60)	176.30 (145.70, 243.65)
Elbow flexors[a]	Active	184.05 (73.29)	207.98 (66.08)	201.99 (64.13)
	Sham	176.61 (35.27)	207.21 (46.65)	210.54 (28.93)
Wrist extensors[a]	Active	115.90 (47.41)	126.59 (55.01)	132.06 (58.44)
	Sham	101.97 (46.41)	123.76 (42.13)	136.55 (48.67)
Elbow extensors[b]	Active	105.10(74.00, 146.40)	117.70 (84.50, 146.40)	114.55 (87.05, 180.10)
	Sham	113.85 (80.20, 145.90)	135.85 (67.50, 153.15)	143.05 (84.00, 164.50)
Wrist flexors[a]	Active	95.98 (53.60)	106.77 (56.42)	111.06 (57.38)
	Sham	82.80 (45.51)	104.01 (49.73)	113.93 (51.03)
Ipsilateral (ipsilateral limb to stimulation brain)				
Shoulder flexors[a]	Active	134.54 (53.25)	149.35 (52.79)	150.00 (55.64)
	Sham	144.23 (41.26)	165.21 (49.71)	177.27 (46.55)
Shoulder abductors[a]	Active	118.41 (51.46)	135.45 (46.02)	140.14 (51.30)
	Sham	134.15 (35.14)	159.23 (44.50)	164.05 (38.23)

Shoulder extensors[a]	Active	112.92 (52.33)	124.58 (52.62)	131.47 (57.38)
	Sham	107.50 (32.24)	133.92 (30.48)	134.85 (35.10)
Shoulder adductors[a]	Active	134.03 (58.14)	156.07 (63.48)	166.98 (65.21)
	Sham	148.87 (41.43)	178.00 (49.03)	184.16 (48.87)
Elbow flexors[a]	Active	175.48 (68.57)	202.36 (71.49)	200.46 (67.85)
	Sham	185.39 (49.77)	214.83 (37.30)	216.73 (44.62)
Wrist extensors[a]	Active	113.73 (53.53)	121.81 (52.48)	132.92 (61.07)
	Sham	112.72 (42.16)	132.95 (41.06)	141.62 (46.13)
Elbow extensors[a]	Active	112.25 (66.55)	121.77 (61.29)	126.24 (68.41)
	Sham	121.72 (42.44)	129.53 (45.04)	140.81 (54.53)
Wrist flexors[a]	Active	94.99 (48.52)	114.61 (58.62)	113.60 (59.31)
	Sham	105.44 (44.03)	125.21 (50.52)	122.42 (53.66)

Note: Higher scores indicate better muscle strength performance.

[a]Data was presented as mean (SD), [b]Data was presented as median (Q1, Q3).
